# Primary caregivers’ perceptions, needs, and expectations regarding nutritional management of children with chronic kidney disease: a qualitative study

**DOI:** 10.3389/fpubh.2026.1822018

**Published:** 2026-05-26

**Authors:** Diyi Luo, Yujia Guan, Lili Liu, Yanni Zheng

**Affiliations:** 1 Department of Pediatric Urinary Disease Center Nursing, West China Second University Hospital, Sichuan University, Chengdu, Sichuan, China; 2 Key Laboratory of Birth Defects and Related Diseases of Women and Children (Sichuan University), Ministry of Education, Chengdu, Sichuan, China; 3 Sichuan Provincial Children's Hospital (Sichuan Provincial Children's Medical Center), Meishan, Sichuan, China; 4 West China Second University Hospital (Tianfu), Sichuan University, Meishan, Sichuan, China

**Keywords:** children, chronic kidney disease, nutritional management, primary caregivers, qualitative research

## Abstract

**Background:**

Nutritional management plays a critical role in optimizing growth, delaying disease progression, and improving long-term outcomes in children with chronic kidney disease. However, caregivers’ perceptions, experiences, and support needs regarding nutritional management remain insufficiently understood.

**Methods:**

A qualitative descriptive study was conducted using semi-structured face-to-face interviews between May and July 2025 in a tertiary women’s and children’s hospital in Chengdu, China. Fifteen primary caregivers of children with CKD were recruited through purposive sampling. Data were analyzed using conventional content analysis with the assistance of NVivo 11.0 software. Data collection continued until thematic saturation was achieved.

**Results:**

Four major themes and related subthemes emerged: (1) mismatch between caregivers’ information needs and the current information environment; (2) variability in caregivers’ willingness and ability to engage in nutritional learning; (3) barriers to translating intention into home-based nutritional management; and (4) caregivers’ needs and expectations regarding nutritional management. Caregivers commonly reported limited access to reliable and authoritative nutritional information, difficulties in appraising information quality, and insufficient continuity of healthcare support. Learning engagement varied according to educational level, caregiving burden, and perceived practicality of information, while culturally influenced preferences (e.g., traditional Chinese medicine) also shaped participation in formal education. Despite generally strong motivation, caregivers frequently experienced an intention–behavior gap, struggling to translate knowledge into consistent dietary practices.

**Conclusion:**

Caregivers of children with CKD face substantial challenges in accessing, understanding, and applying nutritional information in daily care. These difficulties appear to be influenced by limitations in informational support, differences in motivation, and practical caregiving capacity. The findings highlight the importance of providing caregivers with clear, practical, and accessible nutritional support that better facilitates long-term home-based nutritional management for children with CKD.

## 1 Background

Chronic kidney disease (CKD) is defined as structural or functional abnormalities of the kidneys that persist for at least 3 months and have significant implications for health. It encompasses a spectrum ranging from mild renal impairment to end-stage renal disease (ESRD) (1). In recent years, the burden of pediatric CKD has continued to increase, emerging as a major global public health concern. Epidemiological data indicate that the global prevalence of CKD stages 1–5 is 13.4%, with the prevalence of advanced CKD (stages 3–5) reaching 10.6% (2). Further evidence shows a sustained upward trend in pediatric CKD, with the incidence rising from 33.68 per 100,000 in 1990 to 38.11 per 100,000 in 2019, and the prevalence increasing from 412.99 to 483.60 per 100,000 over the same period (3). Compared to adults, children with CKD tend to experience more rapid disease progression and are more susceptible to multisystem complications, including cardiovascular and pulmonary involvement, leading to a significantly increased risk of disability and mortality (4).

Nutritional imbalance is a key factor influencing growth, disease progression, and adverse outcomes in children with CKD (5). In recent years, clinical practice guidelines have been proposed to address the nutritional needs and dietary management of children with CKD stages 2–5, particularly in the dialysis and post-transplantation phases. Vitamin status management has been identified as a critical factor in maintaining the health of these patients, with recommendations for regular monitoring of vitamin levels and personalized supplementation (6). Similarly, the management of potassium, calcium, and phosphate is essential, as both excess and deficiency in these nutrients can adversely affect cardiovascular and skeletal health. Clinical recommendations suggest tailoring potassium intake based on the patient’s condition and maintaining calcium-phosphate balance to prevent bone mineral metabolism disorders (7, 8). Moreover, energy and protein requirements are pivotal for the growth and development of children with CKD. Dialysis patients, in particular, require adequate nutritional support through dietary management to promote growth and tissue repair (9). Another major concern is the high prevalence of obesity and metabolic syndrome in children with CKD, which further exacerbates the disease burden. Clinical guidelines recommend comprehensive interventions, including dietary modifications, physical activity, and pharmacological treatment, to manage obesity and improve metabolic health (10). Thus, accurate assessment and effective management of nutritional status are of paramount importance in pediatric CKD care. Nutritional management in pediatric CKD should be comprehensive and individualized, addressing multiple aspects such as vitamin and mineral status, energy and protein intake, and metabolic abnormalities. This approach ensures optimal nutritional support during treatment, reduces complications, and enhances the quality of life for affected children.

Despite the growing recognition of the importance of nutrition in CKD management, research on caregivers’ nutritional management awareness in China remains limited. Available evidence consistently shows inadequate levels of nutrition-related knowledge among patients and their families. Studies in adult populations have reported that fewer than 50% of patients demonstrate adequate nutritional knowledge, accompanied by poor adherence and insufficient self-management capacity (11–13). In pediatric settings, deficiencies in caregivers’ nutritional knowledge appear even more pronounced. One study found that 76.7% of parents lacked adequate understanding of disease-related nutritional management (14). These findings suggest that caregivers of children with CKD commonly face challenges related to insufficient knowledge, difficulties in implementing nutritional management, and poor sustainability of nutritional practices.

Most existing studies rely on quantitative cross-sectional surveys with limited scope, which restrict their ability to capture caregivers’ lived experiences and explore the underlying reasons for difficulties encountered during nutritional management. Consequently, there is a clear need for qualitative research to comprehensively understand caregivers’ perceptions, challenges, and educational needs related to nutritional management in pediatric CKD.

This study aims to explore in depth the knowledge, needs, and expectations of primary caregivers of children with CKD regarding nutritional management, identify existing problems and barriers within family-based nutritional practices, and provide a theoretical and practical foundation for the development of individualized, caregiver-centered nutritional management interventions.

## 2 Methods

### 2.1 Study design

This study adopted a qualitative descriptive design, informed by a naturalistic and constructivist perspective, to explore primary caregivers’ perceptions, needs, and expectations regarding the nutritional management of children with CKD. This perspective assumes that reality is shaped by individuals’ experiences and interpretations, making it appropriate for understanding caregivers’ views in real-world clinical contexts.

Qualitative descriptive research is particularly suitable when the aim is to obtain a rich, straightforward, and practice-relevant account of participants’ experiences while remaining close to their original expressions. This approach was chosen because the objective of this study was to describe caregivers’ nutrition-related perceptions, support needs, and expectations, rather than to generate new theory (as in grounded theory) or to provide a highly interpretive analysis of lived experience (as in phenomenology).

Accordingly, data were collected through face-to-face semi-structured interviews and analyzed using conventional content analysis, which is well aligned with qualitative descriptive research.

### 2.2 Study setting

The study was conducted at a tertiary women’s and children’s hospital in Chengdu, Sichuan Province, China, between May and July 2025.

### 2.3 Participants and sampling

A purposive sampling strategy was used to recruit primary caregivers of children with CKD. Participants were eligible if they met the following criteria: (1) they were parents or primary caregivers of children with CKD; (2) they had provided daily care for at least 8 h per day for a minimum of 3 months; and (3) they had no diagnosed mental illness and were able to communicate effectively.

Participants were excluded if they were unable to clearly express their views, refused audio recording, withdrew consent prior to the interview, or discontinued participation during the interview process.

Sample size was guided by the principle of data saturation rather than being predetermined in advance. Recruitment, data collection, and preliminary data analysis were conducted iteratively and concurrently. Throughout the data collection process, the research team continuously evaluated whether new themes or meaningful insights were still emerging from the interviews.

Data saturation was considered to have been achieved when no new themes or substantive insights were identified in two consecutive interviews. At this point, participant recruitment was discontinued.

### 2.4 Development of the semi-structured interview guide

The semi-structured interview guide was developed based on a review of relevant domestic and international literature and the researchers’ clinical experience, followed by multiple discussions within the research team. Two caregivers were then invited to participate in pilot interviews. Based on feedback from the pilot interviews, the wording and content of the guide were further refined, resulting in the final version.

The final interview guide covered five domains: (1) knowledge and experiences related to nutritional management; (2) attention to and access to nutrition-related information; (3) difficulties and challenges in nutritional management; (4) needs for nutritional management support; and (5) expectations for future nutritional management. During the formal interviews, the researchers used the guide flexibly and asked probing or follow-up questions as appropriate to facilitate in-depth exploration of participants’ experiences and perspectives.

### 2.5 Data collection

Data were collected through face-to-face semi-structured interviews conducted in a quiet, private, and comfortable office within the hospital. All interviewers had received formal training in qualitative research methods and interviewing techniques and were not involved in the participants’ direct clinical care, thereby reducing the potential for role conflict.

Before each interview, participants were informed of the study objectives, procedures, and potential risks. Participation was entirely voluntary, and written informed consent was obtained from all participants prior to the interviews. During the interviews, the researchers followed the semi-structured interview guide while maintaining flexibility and using probing questions to elicit detailed accounts of participants’ experiences, perceptions, and feelings. With participants’ permission, all interviews were audio-recorded, and field notes were taken to capture non-verbal cues and contextual information, such as facial expressions, tone of voice, and pauses.

Each interview lasted approximately 20–40 min, depending on the depth and richness of the discussion. Audio recordings were transcribed verbatim within 24 h by two researchers independently, and the transcripts were cross-checked for accuracy and completeness. All identifying information was removed and replaced with numerical codes to ensure anonymity. The data were stored in encrypted files accessible only to the research team. Data collection proceeded iteratively and continued until data saturation was achieved.

### 2.6 Researcher reflexivity

The first author was a pediatric nephrology nurse at the time of the study, and the interviews were conducted by trained pediatric nephrology nurses who were familiar with the clinical context of children with CKD. This professional background facilitated communication with participants and supported a contextualized understanding of caregivers’ experiences. At the same time, the research team recognized that such familiarity could also introduce prior assumptions and potential bias during data collection and interpretation.

To minimize interviewer influence and role-related bias, all interviewers had received formal training in qualitative research and interviewing techniques and were not responsible for the participants’ routine clinical management. This helped reduce potential power imbalance and social desirability bias during the interviews. In addition, reflexive discussions were conducted throughout data collection and analysis to critically examine how the researchers’ professional backgrounds and assumptions might influence questioning, coding decisions, and interpretation of the findings. A qualitative methods expert was also involved in reviewing coding and theme development to enhance reflexivity, credibility, and trustworthiness.

### 2.7 Data analysis

A conventional content analysis approach was used to analyze the qualitative data. This analytic method is consistent with the qualitative descriptive design, as it enables researchers to summarize participants’ accounts and organize them into themes and subthemes while remaining close to the original data.

First, all transcripts were read repeatedly to achieve immersion in the data and to gain a comprehensive understanding of participants’ narratives. Meaningful units, key phrases, and statements relevant to the research objectives were identified and coded openly. NVivo software (version 11.0; QSR International, Melbourne, Australia) was used to facilitate data organization and code management. Similar or conceptually related codes were compared iteratively and grouped into preliminary categories. Through continuous comparison with the original transcripts, these categories were further abstracted and refined into overarching themes and subthemes.

To enhance analytical rigor and credibility, two researchers independently conducted the initial coding and theme development. Discrepancies were resolved through discussion and consensus. When necessary, a third qualitative research expert was consulted to achieve agreement and ensure the trustworthiness of the findings. Representative quotations were selected to support each theme and preserve participants’ voices. In addition, reflexive discussions were conducted throughout the analytic process to critically examine coding decisions and interpretation of the data.

This study was conducted and reported in accordance with the Consolidated Criteria for Reporting Qualitative Research (COREQ) checklist, which has been provided as supplementary material. Overall, the study design, data collection, and analysis were aligned with the research objective to ensure methodological congruence.

### 2.8 Ethical considerations

This study was approved by the Ethics Committee of West China Second University Hospital, Sichuan University. As this study involved human participants, all participants were informed of the study objectives, procedures, and potential risks before enrollment. Participation was entirely voluntary, and written informed consent was obtained from all participants prior to the interviews. Confidentiality and anonymity were ensured throughout the study.

## 3 Results

### 3.1 Participant characteristics

A total of 15 primary caregivers of children with CKD were included in this study. The demographic and socioeconomic characteristics of the caregivers, as well as the clinical characteristics of the children, are summarized in Table 1.

**Table 1:** Demographic and clinical characteristics of study participants (*n* = 15).

Caregivers									Patients				
Number	Gender	Age (years)	Residence	Relationship	Education	Monthly income (RMB)	Medical insurance	Family structure	Gender	Age (years)	CKD Staging	Comorbidities	Previous nutritional intervention
R1	Female	30–39	Urban	Mother	College/University	3,000-5,000	Urban resident	Nuclear family	Male	8	G1	Acute pancreatitis	Yes
R2	Male	30–39	Rural	Father	College/University	1,000-2,000	New rural cooperative	Single-parent family	Female	4	G2	Renal hypertension	No
R3	Female	30–39	Urban	Mother	College/University	6,000-8,000	Urban resident	Nuclear family	Male	4	G1	CKD-MBD	No
R4	Male	40–49	Urban	Father	College/University	1,000-3,000	Urban resident	Nuclear family	Male	12	G1	Renal anemia CKD-MBD	No
R5	Male	50–59	Urban	Father	Primary school or below	2,000-4,000	Urban resident	Nuclear family	Female	13	G3b	Renal hypertension Renal anemia	No
R6	Male	40–49	Rural	Father	Primary school or below	1,000-2,000	New rural cooperative	Single-parent family	Male	13	G1	None	No
R7	Female	50–59	Urban	Grandmother	Senior high school/Vocational school	6,000-8,000	Urban resident	Stem family	Female	9	G1	None	Yes
R8	Male	40–49	Urban	Father	Senior high school/Vocational school	4,000-6,000	Urban resident	Nuclear family	Male	17	G1	CKD-MBD	No
R9	Female	30–39	Urban	Mother	College/University	8,000-12,000	Urban resident	Nuclear family	Female	16	G5	Renal hypertension Renal anemia	No
R10	Female	30–39	Urban	Mother	College/University	2,000-4,000	Urban resident	Skipped-generation family	Female	9	G3b	Renal hypertension Renal anemia	No
R11	Female	30–39	Urban	Mother	Junior high school	1,000-2,000	Urban resident	Nuclear family	Male	11	G1	Acute pancreatitis Renal hypertension Renal anemia	No
R12	Female	20–29	Rural	Mother	Junior high school	2,000-4,000	Self-funded	Nuclear family	Female	8	G5D	Renal hypertension Renal anemia	No
R13	Female	30–39	Rural	Mother	Senior high school/Vocational school	1,000-2,000	New rural cooperative	Nuclear family	Male	9	G5	Renal hypertension Renal anemia	No
R14	Female	30–39	Urban	Mother	Junior high school	1,000-2,000	Urban resident	Single-parent family	Female	4	G5	Renal hypertension Renal anemia	No
R15	Female	30–39	Urban	Mother	College/University	1,000-2,000	Urban resident	Single-parent family	Female	10	G5	Renal hypertension Renal anemia	No

CKD, chronic kidney disease; CKD-MBD, chronic kidney disease–mineral and bone disorder; G1–G5D, chronic kidney disease stages according to KDIGO classification; G5D, stage 5 chronic kidney disease requiring dialysis; RMB, Renminbi.

### 3.2 Overview of themes

**Figure 1:**
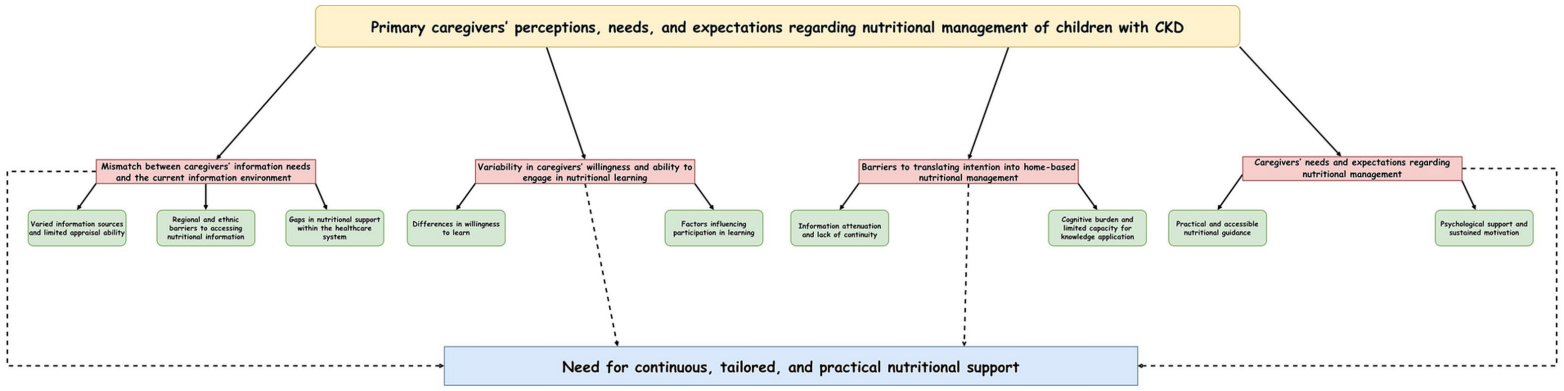
Thematic framework of primary caregivers’ perceptions, needs, and expectations regarding nutritional management in children with CKD. This figure presents the four major themes and corresponding subthemes derived from the qualitative analysis: (1) Mismatch between caregivers’ information needs and the current information environment; (2) variability in caregivers’ willingness and ability to engage in nutritional learning; (3) barriers to translating intention into home-based nutritional management; and (4) caregivers’ needs and expectations regarding nutritional management. Together, these themes highlight the need for continuous, tailored, and practical nutritional support.

A total of four major themes and corresponding subthemes emerged from the data analysis (Figure 1). These themes capture primary caregivers’ perceptions, needs, and expectations regarding the nutritional management of children with CKD, including: (1) mismatch between caregivers’ information needs and the current information environment; (2) variability in caregivers’ willingness and ability to engage in nutritional learning; (3) barriers to translating intention into home-based nutritional management; and (4) caregivers’ needs and expectations regarding nutritional management.

Together, these themes highlight a consistent pattern in which caregivers demonstrate strong motivation but encounter multiple barriers in accessing, understanding, and applying nutritional information, ultimately underscoring the need for continuous, tailored, and practical nutritional support.

### 3.3 Theme 1: mismatch between caregivers’ information needs and the current information environment

This theme describes the gap between caregivers’ strong demand for nutritional information and the limitations of current information sources and healthcare support systems.

#### 3.3.1 Varied information sources and limited appraisal ability

Caregivers obtained nutritional information from multiple channels, including healthcare professionals, nutrition clinics, online platforms, and peer communication. Some caregivers actively integrated information from different sources. For example, R1 stated:

“Whenever we are hospitalized, we consult with the nutrition clinic doctor… I also use my phone to search for information and discuss it with other parents.”

However, most caregivers relied heavily on online information and reported difficulty evaluating its accuracy and reliability. Confusion caused by inconsistent information was common. As R10 explained:

“Some say shrimp can be eaten, others say it can’t, so we really don’t know whether it’s okay or not.”

#### 3.3.2 Regional and ethnic barriers to accessing nutritional information

Caregivers from remote areas or ethnic minority backgrounds faced additional barriers, including language differences, limited access to healthcare resources, and insufficient health education. These challenges restricted their access to reliable nutritional information and resulted in limited understanding. As one caregiver (R13) stated succinctly:

“I don’t know.”

#### 3.3.3 Gaps in nutritional support within the healthcare system

Many caregivers perceived nutritional guidance within the healthcare system as fragmented and lacking continuity. Information provided was often limited to general advice without detailed or practical guidance.

For instance, R7 stated:

“The doctor told me not to eat very spicy food, but I can’t remember much else.”

In addition, insufficient coordination between departments further weakened the effectiveness of nutritional support. As R6 reported:

“The nutrition department arranged a meal for the child, but it was wrong on the first day.”

Some caregivers also noted that they needed to actively seek information rather than receiving proactive guidance from healthcare providers, indicating gaps in the delivery of nutritional education.

### 3.4 Theme 2: variability in caregivers’ willingness and ability to engage in nutritional learning

This theme reflects differences in caregivers’ motivation, engagement, and capacity to participate in nutritional education.

#### 3.4.1 Differences in willingness to learn

Most caregivers expressed a strong willingness to learn, recognizing the importance of nutritional management in disease care. For example, R2 stated:

“Of course it’s necessary.”

Similarly, R11 emphasized:

“If it were scored out of five, I’d give it a full five.”

However, some caregivers demonstrated conditional engagement, depending on the perceived usefulness of the content. As R9 noted:

“If it’s really useful, then I’d definitely go. But if it’s not very useful, I probably wouldn’t.”

A minority of caregivers showed low interest in formal education and preferred alternative approaches. For instance, R4 stated:

“Under the guidance of traditional Chinese medicine… there’s no need to attend other lectures.”

#### 3.4.2 Factors influencing participation in learning

Caregivers’ engagement in learning was shaped by multiple factors, including educational format, time availability, financial burden, and educational background.

Many caregivers preferred clear, practical, and easily accessible educational materials. For example, R11 stated:

“Paper leaflets are better—what can be eaten, what can’t be eaten—something more straightforward.”

Others expressed a preference for flexible delivery methods, such as scheduled training sessions or digital communication. As R8 suggested:

“WeChat push messages would work.”

Time constraints, financial pressure, and lower educational levels further limited participation. As R7 explained:

“I don’t have time. I’m busy working in the fields every day.”

### 3.5 Theme 3: barriers to translating intention into home-based nutritional management

This theme captures the challenges caregivers faced in converting knowledge and intention into sustained dietary practices.

#### 3.5.1 Information attenuation and lack of continuity

Nutritional education was often delivered through one-way communication with limited reinforcement or follow-up, resulting in gradual information loss over time. Many caregivers reported difficulty recalling and applying dietary advice after discharge, reflecting insufficient continuity of education.

In addition, poor coordination among healthcare providers further contributed to gaps in information delivery. As R10 reported:

“The attending doctor said they would ask the nutrition department to consult, but the nutrition doctor never came.”

#### 3.5.2 Cognitive burden and limited capacity for knowledge application

Caregivers frequently experienced difficulty understanding complex nutritional information, which reduced their ability to apply recommendations in daily life. Some caregivers reported disengaging from information that they found difficult to comprehend. As R9 stated:

“I didn’t really understand it, so I didn’t keep looking at it.”

Even caregivers with medical backgrounds encountered challenges in interpreting disease-specific dietary recommendations. As R11 noted:

“Even though I studied medicine, I still can’t figure it out.”

Memory limitations further hindered implementation. As R8 explained:

“I’ve forgotten what foods can’t be eaten.”

### 3.6 Theme 4: caregivers’ needs and expectations regarding nutritional management

Caregivers expressed multi-dimensional needs related to nutritional management, including practical guidance, improved information accessibility, healthcare system support, and psychological assistance.

#### 3.6.1 Practical and accessible nutritional guidance

Caregivers emphasized the need for structured, clear, and actionable dietary guidance, particularly in the early stages of disease management. For example, R1 stated:

“At the beginning, we were very confused… could there be a general framework?”

Accessibility was also a key concern, especially for those in remote areas. Many caregivers preferred ongoing digital support after discharge. As R2 noted:

“When we go home for recovery, information could be pushed through WeChat.”

Caregivers also highlighted the importance of aligning hospital-provided meals with dietary recommendations. For instance, R3 reported:

“We noted ‘less oil, less salt, no chili,’ but the meal delivered was still salty and spicy.”

#### 3.6.2 Psychological support and sustained motivation

Beyond practical needs, caregivers expressed a strong demand for psychological support and sustained motivation, particularly when managing long-term dietary restrictions.

Adolescence was identified as a challenging period for dietary management. As R4 explained: “The child is in adolescence now, and the concepts parents try to instill may not be accepted.”

Caregivers also described difficulties in managing children’s eating behaviors. As R1 noted: “When the food has no salt, he eats very little… but when there’s oil and meat, he eats a lot.”

In addition, caregivers emphasized the importance of professional guidance in strengthening confidence and adherence. As R11 stated: “It gives parents strong confidence.”

## 4 Discussion

### 4.1 Mismatch between caregivers’ information needs and the current information environment

This study revealed a marked mismatch between caregivers’ strong demand for nutritional knowledge and the limitations of the current information environment for pediatric CKD. Although most caregivers expressed a clear willingness to learn, they frequently lacked access to reliable, authoritative, and practical guidance, and were often exposed to fragmented, inconsistent, or low-quality information from online and informal sources. From the perspective of the Information–Motivation–Behavioral Skills (IMB) model, accurate and understandable information is a foundational prerequisite for health-related behavior change. However, our findings suggest that caregivers were often expected to manage complex nutritional issues in the context of insufficient informational support, which may partly explain why strong learning intentions did not readily translate into effective nutritional management behaviors.

This finding is also consistent with a health literacy perspective. Health literacy involves not only obtaining information, but also the ability to access, understand, appraise, and apply that information in everyday decision-making. In the present study, caregivers frequently reported difficulty judging the accuracy and credibility of nutrition-related information, especially when relying on online sources (15, 16). Furthermore, nutritional education provided within the healthcare system was often fragmented, non-systematic, and insufficiently reinforced, with limited multidisciplinary collaboration and follow-up. These challenges were further intensified by geographic barriers, language difficulties, and unequal access to healthcare resources.

Importantly, caregivers reported greater trust in guidance from medical institutions and health professionals and expressed a clear preference for structured, authoritative, and actionable recommendations. This suggests that healthcare systems should play a more proactive role in shaping the caregiver information environment. Integrating evidence-based nutritional guidance into widely used digital platforms and strengthening continuity between in-hospital education and community- or home-based support may help improve the accessibility, credibility, and practical value of nutritional information (17).

### 4.2 Variability in caregivers’ willingness to learn and implications for tailored education

Caregivers’ willingness to learn about nutritional management varied considerably and was shaped by cognitive capacity, cultural background, caregiving burden, competing daily responsibilities, and access to information. Within the IMB framework, motivation represents a key determinant of whether individuals are willing to engage in health-related learning and behavior change. In this study, some caregivers actively sought professional support and demonstrated strong engagement in dietary management, whereas others showed limited participation unless the educational content was perceived as directly relevant, practical, and easy to implement. For some families, limited education, financial pressure, and time constraints further reduced their ability to engage with nutritional education in a sustained way.

Notably, some caregivers expressed a preference for traditional Chinese medicine (TCM)–based approaches over formal nutritional education. This finding reflects the broader cultural context in which TCM is widely accepted and trusted as part of routine health management in China. From a behavioral perspective, such culturally rooted beliefs may influence caregivers’ motivation and engagement in evidence-based nutritional education, and in some cases reduce their willingness to participate in structured learning activities.

These findings suggest that caregivers should not be treated as a homogeneous educational target group. Instead, nutritional support should be tailored according to both motivational readiness and health literacy level. Caregivers with higher motivation may benefit from more comprehensive and systematic educational resources, while those with lower motivation or more limited capacity may require simplified, highly practical, and immediately applicable support. Educational strategies such as concise written materials, visual aids, digital micro-learning resources, and repeated reinforcement may help improve both engagement and comprehension.

At the same time, reliance on TCM should not be interpreted solely as a barrier. Rather, it may represent a culturally grounded coping strategy that provides caregivers with a sense of familiarity, trust, and perceived effectiveness in managing their child’s condition. Therefore, instead of directly opposing TCM beliefs, healthcare providers should adopt culturally sensitive communication strategies that acknowledge caregivers’ perspectives while gradually introducing evidence-based nutritional guidance. Integrating culturally familiar concepts with scientifically grounded recommendations may enhance trust, improve communication, and ultimately facilitate better adherence to nutritional management.

In addition, motivation in the context of chronic pediatric illness is unlikely to be static. Long-term caregiving stress, uncertainty about the child’s prognosis, and repeated exposure to complex dietary recommendations may all weaken sustained participation over time. Therefore, caregiver-centered education should not only deliver information but also acknowledge emotional burden, reinforce confidence, and support continued engagement. Tailored and flexible nutritional education may be particularly important for promoting equitable support across caregiver groups with differing resources and capacities.

### 4.3 Barriers from intention to implementation and directions for improvement

A key finding of this study was the existence of an intention–behavior gap: although many caregivers expressed strong willingness to learn and recognized the importance of nutritional management, they often encountered substantial difficulty in translating this intention into sustained dietary practice at home. This gap can be more effectively interpreted using the Information–Motivation–Behavioral Skills (IMB) model, which emphasizes that behavior change depends not only on adequate information and motivation, but also on the practical skills required to implement health recommendations in everyday life. In the present study, caregivers’ implementation difficulties appeared to arise from the interaction of limited health literacy, inconsistent educational support, caregiving burden, and insufficient opportunities to develop practical management skills.

Lower educational attainment and financial constraints were associated with reduced health information literacy, which may have weakened caregivers’ ability to understand, appraise, and apply dietary recommendations (18–21). In addition, nutrition education was often delivered through one-way communication, with limited reinforcement, visualization, feedback, or skills-based coaching, and was frequently confined to hospitalization periods. The absence of sustained post-discharge support further reduced caregivers’ ability to maintain nutritional management behaviors in the home setting. These findings suggest that the challenge is not merely a lack of willingness, but also a lack of structured support to translate knowledge into feasible daily practice.

Accordingly, improving nutritional management for children with CKD requires more than the provision of information alone. Interventions should address all three dimensions highlighted by the IMB model: providing clear and trustworthy information, sustaining caregiver motivation under long-term caregiving demands, and strengthening practical behavioral skills such as meal planning, food selection, portion adjustment, and problem-solving within family routines. A multimodal and continuous nutritional education model may therefore be particularly beneficial. Combining in-hospital guidance with post-discharge follow-up, and integrating oral, written, visual, and digital resources, could help caregivers better understand, retain, and apply nutritional recommendations in daily life. Such approaches may enhance caregivers’ health literacy and self-efficacy, thereby facilitating the translation of knowledge into consistent caregiving practices and improving adherence to nutritional management in children with CKD (22–24).

In summary, caregivers of children with CKD often showed strong willingness to support dietary management but experienced considerable difficulty implementing nutritional recommendations consistently in daily life. From the perspectives of the IMB model and health literacy, these challenges appear to arise from the combined effects of limited informational support, variability in motivation, and insufficient practical management skills. These findings highlight the need for more structured and feasible nutritional support for caregivers in long-term home-based care.

## 5 Conclusion

This study provides insight into the experiences, perceptions, and support needs of primary caregivers involved in the nutritional management of children with CKD. Although most caregivers demonstrated strong motivation to support dietary management, they faced substantial challenges in accessing reliable information, understanding nutritional guidance, and applying recommendations consistently in daily life.

Interpreted through the perspectives of health literacy and the IMB model, these findings suggest that nutritional management difficulties are influenced not only by informational limitations, but also by differences in motivation and practical caregiving skills. In addition, culturally shaped health beliefs may influence caregivers’ engagement with nutritional education and should be considered in caregiver support.

Overall, the findings highlight the importance of providing caregivers with clear, practical, and accessible nutritional guidance that better supports long-term home-based management for children with CKD.

## 6 Limitations

This study has several limitations. First, although data saturation was achieved, the determination of saturation in qualitative research is inherently interpretive, and researchers’ perspectives may have influenced data interpretation. To enhance analytical rigor, independent coding and reflexive discussions were conducted, and a qualitative methods expert reviewed the analytic process.

Second, although efforts were made to promote openness and confidentiality during the interviews, social desirability bias cannot be entirely excluded. As the interviews were conducted within a hospital setting by researchers affiliated with the same institution, some participants may have provided responses that they perceived as socially acceptable or consistent with clinical expectations. In addition, interviewer characteristics may have influenced data collection. Although the interviewers were trained in qualitative methods and were not directly involved in participants’ routine clinical care, their professional background in pediatric nephrology may have shaped both the interview process and participants’ responses.

Third, all participants were recruited from a single tertiary hospital, which may limit the transferability of the findings to other healthcare settings or regions with different medical resources and sociocultural contexts. In addition, the participating children presented with considerable clinical heterogeneity, including differences in CKD stage, comorbidities, and previous exposure to nutritional interventions. The sample included children ranging from early-stage CKD to dialysis-dependent disease, with varied accompanying health conditions and nutritional management experiences. Although such diversity may enrich the range of caregiver perspectives captured in qualitative research, these differences may also have influenced caregivers’ perceptions, informational needs, and expectations regarding nutritional management. Therefore, caution should be exercised when considering the transferability of the findings to other populations and clinical contexts.

Finally, a relatively high proportion of caregivers in this study did not have medical backgrounds. Although this may limit the representation of medically trained caregivers, it also reflects the perspectives of lay caregivers who are primarily responsible for day-to-day nutritional management in real-world settings.

Future studies should expand recruitment across multiple centers and include caregivers with more diverse cultural, educational, and professional backgrounds. In addition, mixed-methods research may help to further validate and extend these findings. The development and evaluation of stratified and caregiver-tailored nutritional education interventions may provide more targeted and practical support for children with CKD and their families.

## Data Availability

The original contributions presented in the study are included in the article/supplementary material, further inquiries can be directed to the corresponding author.
